# 24-hour urinary total protein quantitative detection for pregnant women with unit conversion failure: a case report and laboratory administration reflection

**DOI:** 10.3389/fmed.2026.1763851

**Published:** 2026-03-16

**Authors:** Guangjun Xiao, Yanting Liu, Juan Hu, Bin Peng, Weifeng Liao, Huanhuan Wang, Shaocheng Zhang

**Affiliations:** 1 Department of Clinical Laboratory, Suining Central Hospital, Suining, Sichuan, China; 2 School of Laboratory Medicine, Chengdu Medical College, Chengdu, Sichuan, China; 3 Clinical IVD Joint Research Center of Chengdu Medical College-Maccura Biotechnology, Chengdu, Sichuan, China; 4 Department of Laboratory Medicine, The Second Affiliated Hospital of Chengdu Medical College (Nuclear Industry 416 Hospital), Chengdu, Sichuan, China; 5 Irradiation Preservation and Effect Key Laboratory of Sichuan Province, Chengdu, Sichuan, China

**Keywords:** 24-hour urinary total protein quantification, high-concentration sample, Laboratory Information System, laboratory quality management, unit conversion

## Abstract

Analogous to medication dosage errors, inaccuracies in measurement units for analyte test results in clinical laboratories can lead to adverse clinical consequences. We report a case of a 35-years-old pregnant woman at 38 weeks of gestation with a history of gestational hypertension. During routine follow-up, she presented with elevated blood pressure, abnormal qualitative urinary protein findings, and an elevated random urinary protein concentration, prompting a request for 24-hour urinary total protein quantification (UTP). The laboratory reported her 24-hour urine total protein excretion as 547.87 g/24 h, which the attending physician identified as inconsistent with the patient’s clinical manifestations. Unfortunately, the medical laboratory professional failed to identify the cause of the discrepancy following receipt of the feedback and only reported the issue to the laboratory quality supervisor 1 week later. In the detection system, both the direct and 1:2 auto-diluted measurements triggered an “F” alarm code, indicating the analyte concentration exceeded the analytical measurement range. When the alarm code in the data flag column and the analyte measurement result were simultaneously transmitted to the Laboratory Information System (LIS), precluding accurate data recognition and extraction by the LIS. This led to the appearance of the alarm code in the LIS result field and disrupted the automatic unit conversion process. According to relevant requirements (such as those in the detection system operation manual or the laboratory-developed standard operating procedure), if the alarm code appears in the data flag column of the measurement result, it is necessary to further increase the dilution ratio and conduct a re-measurement. Regrettably, the alarm code was mishandled in the laboratory: it was manually suppressed in the LIS. This resulted in the failure of the automatic unit conversion, leading to a 100-fold overestimation as the results were erroneously reported in “g/L” instead of mg/dL.” This case highlights critical quality management considerations for other clinical laboratories, emphasizing the importance of unit consistency across diverse detection platforms and information systems, and the quality risks associated with falsely decreased results when analyzing high-concentration samples.

## 1 Introduction

Hypertensive disorders of pregnancy (HDP) are among the most common pregnancy complications and a leading cause of maternal mortality worldwide, posing a severe threat to maternal health and significant risks to fetal and neonatal outcomes (1). In recent years, the incidence of HDP has risen significantly alongside the increasing prevalence of risk factors such as advanced maternal age, obesity, and diabetes, which undoubtedly exacerbates the global burden of disease (1). Consequently, the prevention, early diagnosis, and prompt management of HDP are of paramount importance. Since women with HDP typically present with hypertension, proteinuria, and thrombocytopenia (1), non-invasive screenings–including regular blood pressure monitoring, qualitative urinalysis, microalbuminuria testing, and the urinary albumin-to-creatinine ratio (uACR)–are routinely employed for early detection within comprehensive maternal health management (2). If qualitative tests on morning or random urine samples yield positive results, 24-hour urinary total protein quantification (UTP) is frequently performed to further assess maternal status and the clinical risk of HDP (2). Consequently, ensuring the reliability and accuracy of 24-hour UTP results is of critical significance in the clinical management of pregnant women.

Laboratory test results play a crucial role in disease diagnosis and the development of treatment strategies. However, the accurate interpretation of test results also relies on the correct application of units of measurement (3). Errors in the use of units for analyte test results may directly undermine the accuracy of clinical decision-making, thereby leading to adverse clinical outcomes (3). The units of measurement for the original analyte results from a laboratory detection system are often determined by the units of the calibrator. Nevertheless, the units used in test reports must align with clinical guidelines and expert consensus to facilitate accurate clinical application of laboratory results. This may result in discrepancies between the units of the detection system’s original measurement results and those in the final test reports. Unfortunately, this is the case for the 24-hour UTP test routinely conducted in clinical laboratories. Clinical practice shows that some clinical laboratories still establish automatic unit conversion procedures in the Laboratory Information System (LIS), which reconcile units between the detection system and the LIS by multiplying the received original results by a conversion factor. Errors in automatic unit conversion may occur if Errors in data transmission of analyte measurement results from the detection system, or incorrect parsing/abnormal value extraction of measurement data by the LIS, the conversion factor is set incorrectly, or modifications are made to the detection system’s measurement procedures or the LIS–all of which increase risks to patients.

In this report, we describe a case where failed unit conversion led to a 24-hour urinary total protein quantification result that was inconsistent with both the patient’s clinical manifestations and medical common sense. This case highlights the importance of unit consistency across different detection platforms or information systems, and the quality risks associated with falsely decreased results when non-matched detection systems–constructed from various combinations of analyzers, calibrators, and reagents from different manufacturers–are used to measure high-concentration samples exceeding the Analytical Measurement Range (AMR). It also provides guidance for quality improvement in other clinical laboratories.

## 2 Case report

A 35-years-old pregnant woman at 38 weeks of gestation with a history of gestational hypertension presented for a routine follow-up visit. Her blood pressure was measured at 167/84 mmHg, and the qualitative urinary protein test result was “+++” (reference range: “−”). The random urinary total protein concentration was 2.84 g/L (reference range: <0.15 g/L), the creatinine concentration was 4638.6 μmol/L (Table 1), and the urinary total protein/creatinine ratio was 5.41 g/g (reference range: <0.2 g/g) [urinary total protein and creatinine were detected using an AU5800 analyzer (Beckman Coulter, Inc., Fullerton, CA, USA); CREA reagents and calibrators were sourced from Beckman Coulter, Inc., while urinary total protein quantification reagents and calibrators were obtained from Beijing Leadman Biochemical Co., Ltd., Beijing, China]. The physician ordered a 24-hour urinary total protein quantification test for further clinical evaluation. The pregnant woman collected all urine produced over a 24-h period, with a total volume of 1200 mL, and submitted it to the laboratory during the night for analysis. The laboratory report provided to the ordering physician (Table 1) showed a 24-hour urinary total protein concentration of 456.56 g/L (reference range: <0.15 g/L), corresponding to a 24-hour urinary total protein excretion of 547.87 g/24 h (reference range: <0.15 g/24 h). Compared with the random urinary total protein concentration, the 24-hour urinary total protein concentration exhibited a striking 161-fold discrepancy. Consequently, the ordering physician contacted the laboratory to inquire about the potential cause of the abnormally high 24-hour urinary total protein value.

**TABLE 1:** Laboratory test results and dilution test comparison for the pregnant woman.

Analyte	Pre-adjustment of the dilution ratio in the automatic dilution procedure				Result of the 24-hour urine sample re-measurements post 1:10 automatic dilution
	Random urine result	Direct measurement result of the 24-h urine sample (no dilution)	Result of the 24-h urine sample processed by automatic dilution procedure (1:2 dilution)	Result of the 24-h urine sample after re-measurement using the automatic dilution procedure (1:2 dilution)	
UTP (g/L)	2.84^c^	376.43^a,d^	456.56^a,b,c^	460.60^a,b,d^	5.30^e^
Crea (μmol/L)	4638.6^c^	N/A	N/A	N/A	N/A
24-hour Total Urine Volume (mL)	N/A	1200	1200	1200	1200
24h UTP (g/24 h)	N/A	N/A	547.87^b,c^	552.72^b,d^	6.34

Crea, creatinine; UTP, urinary total protein quantitative; 24h UTP, 24-hour urine total protein. *^a^*Measurement results were marked with the alarm code “F.” *^b^*Results released after deleting the alarm code. *^c^*Analyte measurement results received by the requesting physician. *^d^*Measurement results were used for internal laboratory verification only and were not reported to the requesting physician. *^e^*Measurement results lacked the alarm code “F.”

After receiving feedback from the requesting physician, the medical laboratory professional checked the measurement results of the analyte in the detection system. It was found that the sample underwent direct measurement and automatic dilution measurement sequentially in the detection system. The data displayed in the measurement result column were 376.43 and 456.56 respectively, and the alarm code “F” appeared in the data flag column for both measurements (This alarm code indicates that the measurement result of the analyte exceeds the Analytical Measurement Range of the detection system) (Table 1). The automatic dilution measurement procedure was rerun on the pregnant woman’s sample, and the data in the measurement result column showed 460.60, with the alarm code “F” still present in the data flag column (Table 1). After the detection system completed the measurement of the analyte, the measurement results transmitted to the LIS were displayed as “376.43F” and “456.56F” respectively. According to the laboratory-developed standard operating procedure (SOP), when the alarm code “F” appears, it is required to further increase the dilution ratio and conduct a re-measurement. However, regrettably, the medical laboratory professional did not pay sufficient attention to the alarm code in the LIS result field. They might have simply assumed that the code was due to abnormal data extraction in the LIS and failed to strictly follow the prompts in the operation manual of the detection system or the SOP to increase the dilution ratio and re-measure. When comparing with the initial automatic dilution result, it was found that the percentage difference between the retest result and the initial value was 0.88%, which is far below the laboratory’s analytical performance criteria (Total Allowable Error, TEa: target value ± 30%; acceptable percentage difference < 1/3 TEa, i.e., <10%). Therefore, the medical laboratory professional concluded that the two test results were consistent. Instead of properly addressing the alarm code as required by the SOP, they removed the alarm code “F” from the LIS result field. Prior to the release of this test report, if the medical laboratory professional had rigorously performed delta checks and conducted a comparative discrepancy analysis with the patient’s random urinary total protein results in the LIS, they might have identified the unit mismatch between the detection system and the LIS in a timely manner, and potentially identified the root cause of the LIS-based automatic unit conversion failure, thereby preventing the issuance of this erroneous report to the ordering physician.

Notably, it was not until 1 week later that medical laboratory professional acknowledged the potential inaccuracy of the patient’s 24-hour UTP result and escalated the issue to the quality supervisor. They requested a formal investigation into the root cause to implement corrective measures and prevent recurrence.

## 3 Laboratory evaluation

### 3.1 Laboratory investigation

After receiving feedback from the medical laboratory professional, we initially verified the patient’s test report within the LIS (Table 1). The analyte results within the LIS were consistent with those displayed in the Hospital Information System (HIS), thus ruling out errors arising from abnormal data transmission between the two systems. We conducted a further investigation into pre-analytical errors, including errors in sample information, inappropriate sample types, and insufficient mixing during sample aliquoting. Following the exclusion of these factors, we focused our investigation on in-laboratory analytical processes that could impact the accuracy of test results. A comprehensive assessment–incorporating feedback from External Quality Assessment (EQA) programs [organized by the National Center for Clinical Laboratories (NCCL)], internal quality control (IQC) data, and clinical feedback on other patients’ test results–confirmed the normal operation of the detection system. However, when medical laboratory professionals reviewed the measurement parameters for the analyte within the detection system and the calibrator instructions, they found that the calibrator’s labeled concentration was expressed in “mg/dL.” The laboratory directly utilized the calibrator’s labeled concentration for analyte calibration to generate the calibration curve, resulting in the patient’s sample test results being reported in “mg/dL” within the detection system–a unit inconsistent with the “g/L” employed in the LIS.

To convert the unit of the analyte to “g/L” (a unit commonly used in clinical practice in China), the laboratory implemented an automatic unit conversion procedure within the LIS. Specifically, the LIS automatically multiplied all original UTP results by 0.01 (conversion factor: 1 g/L = 100 mg/dL) and subsequently displayed the analyte concentration with the converted unit within the LIS user interface. This process was designed to achieve unit consistency between the detection system and the LIS.

An additional review of the original analyte results within the detection system and the reported results within the LIS revealed that the patient’s sample exhibited the alarm code “F” alongside its original results across all three tests: the initial test, the automatic dilution test, and the retest following the physician’s feedback (Table 1). In contrast, no alarm code was present alongside the original results for other samples. Following the transmission of the original results to the LIS, the automatic unit conversion procedure was triggered for all samples except for the patient’s sample. Therefore, the abnormally high analyte result for the pregnant woman was likely attributed to the alarm code “F” present in the original results from the detection system.

### 3.2 Experimental verification

A serum sample with a total protein concentration of 74.06 g/L was diluted with physiological saline to generate four validation samples at predefined dilution ratios of 1:8, 1:9, 1:10, and 1:37. After mixing, the UTP detection procedure was employed to determine the total protein concentration for these four diluted serum samples. Except for Sample 4, all other samples exhibited the alarm code “F” in their respective measurement results; furthermore, the automatic unit conversion procedure within the LIS failed to be triggered for these results following their transmission to the LIS (Table 2). This finding indicated that when an original measurement result was accompanied by the alarm code “F,” it will impact LIS data extraction. As a result, the alarm code shows up in the LIS result field, causing the automatic unit conversion procedure to fail. Ultimately, the analyte results within the LIS and test reports were not multiplied by the corresponding conversion factor during unit conversion, leading the attending physician to misinterpret the analyte concentration as 100 times higher than the actual concentration.

**TABLE 2:** Differences between measured results and theoretical values for high-concentration samples.

Sample No.	Theoretical value (mg/dL)	Direct measurement (no dilution)			Automatic dilution (1:2)			Automatic dilution (1:10)			Quality requirement for PD (%)
		Measured value (mg/dL)	PD (%)	LIS-displayed value (g/L)	Measured value (mg/dL)	PD (%)	LIS-displayed value (g/L)	Measured value (mg/dL)	PD (%)	LIS-displayed value (g/L)	
1 (1:8 dilution)^a^	925.7	390.4^b^	−57.83^d^	390.4F^e^	583.5^b^	−36.97^d^	583.5F^e^	807.3^c^	−12.80	8.07^f^	−15 15
2 (1:9 dilution)^a^	822.9	379.8^b^	−53.84^d^	379.8F^e^	552.6^b^	−32.85^d^	552.6F^e^	717.0^c^	−12.86	7.17^f^	−15 15
3 (1:10 dilution)^a^	740.6	372.5^b^	−49.70^d^	372.5F^e^	535.2^b^	−27.73^d^	535.2F^e^	691.2^c^	−6.66	6.91^f^	−15 15
4 (1:37 dilution)^a^	200.2	144.2^c^	−27.94^d^	1.44^f^	163.3^c^	−18.41^d^	1.63^f^	173.2^c^	−13.46	1.73^f^	−15 15

LIS, Laboratory Information System; PD, percentage difference. *^a^*Sample prepared by diluting serum with saline at specified dilution ratios. *^b^*Measurement results in the detection system were marked with the alarm code “F.” *^c^*No alarm code “F” was marked for measurement results in the detection system. *^d^*The PD between the measured result and the theoretical value did not meet the laboratory’s quality requirements. *^e^*The LIS did not automatically execute the unit conversion procedure after receiving the measurement result. *^f^*The LIS automatically executed the unit conversion procedure after receiving the measurement result.

Notably, when the four samples with distinct total protein concentrations were measured directly via the detection system (i.e., without dilution), the percentage difference between the measured results and the theoretical values ranged from −27.94% to −57.83%, which was unacceptable as it did not meet the laboratory’s quality criteria (TEa: target value ± 30%; acceptable percentage difference < 1/2 TEa, i.e., <15%). When the automatic dilution procedure (set to a 1:2 dilution ratio) was employed, the percentage difference between the measured results and the theoretical values decreased to between −18.41% and −36.93%. Although this represents an improvement compared to the non-diluted measurement, the results still failed to meet the laboratory’s quality criteria (Table 2).

This discrepancy can be attributed to multiple factors. Firstly, the analyte concentration in the samples may have exceeded both the Analytical Measurement Range (0–200 mg/dL) and the Clinically Reportable Range (CRR; 0–400 mg/dL) of the detection system. Secondly, the 1:2 dilution ratio set in the automatic dilution procedure may have been inadequate. On the basis of the results from the analyte dilution and recovery test (Supplementary Table 1), the dilution ratio in the automatic dilution procedure was adjusted from 1:2 to 1:10. The four high-concentration samples were subsequently retested, and the results demonstrated that the percentage difference between the measured analyte values and the theoretical values was less than 1/2 TEa, thus meeting the laboratory’s quality criteria (Table 2). This finding confirms that the appropriateness of the dilution ratio in the automatic dilution procedure significantly impacts the accuracy of test results for high-concentration samples. Therefore, laboratories should select an appropriate dilution ratio on the basis of evaluation results from dilution and recovery tests, and dynamically adjust it in accordance with the analyte concentration range in the served population, to ensure the adequacy of the detection system’s CRR.

### 3.3 Patient sample retesting

Following the adjustment of the dilution ratio in the automatic dilution procedure to 1:10, the pregnant woman’s sample was retested. The measured result exhibited a UTP concentration of 530.2 mg/dL with no accompanying alarm code “F.” Following the transmission of this result to the LIS and subsequent execution of the automatic unit conversion procedure, the UTP result was displayed as 5.30 g/L (Table 1). This corresponded to a 24-hour urinary total protein excretion of 6.34 g/24 h (reference range: <0.15 g/24 h). When compared to the manually converted 1:2 dilution rerun result, although the percentage difference in UTP result was only 14.97% (Table 1), adjusting the dilution ratio expanded the Clinically Reportable Range (0–20 g/L) of the detection system, thereby reducing the risk of failure in the automatic unit conversion procedure.

## 4 Discussion

As the final output of routine testing activities in clinical laboratories, test reports derive their core value from the full and accurate presentation of all information necessary for interpreting analyte measurement results (4–6). Clause 7.4.1.6 of ISO 15189:2022 (Medical Laboratories – Requirements for Quality and Competence) specifies in detail the information to be included in test reports and explicitly recommends that the units of measurement for analyte results should preferably be expressed in International System of Units (SI) or units traceable to SI (4). However, feedback from clinical practice indicates that unit conversion of some analyte results remains necessary prior to report release–for example, conversion between amount-of-substance concentration units (e.g., mol/L) and mass concentration units (e.g., g/L), or between commonly used clinical units and SI units (3).

The 24-hour urinary total protein excretion is a key biomarker for the diagnosis, management, and efficacy monitoring of kidney diseases (7, 8). In clinical practice in China, the commonly used units for this analyte are typically g/L or g/24 h. In this case, the calibrator of the laboratory’s analyte measurement kit was calibrated in “mg/dL,” resulting in the detection system reporting analyte results in “mg/dL”–a unit inconsistent with clinically used units. Although the laboratory recognized this discrepancy and implemented an automatic unit conversion procedure within the LIS to align results and units between the detection system and the LIS, it presumably overlooked the impact of alarm codes (included in the detection system’s results) on LIS data extraction during data transmission verification. This resulted in the appearance of the alarm code in the LIS result field and disrupted the functionality of the automatic unit conversion procedure. This indicates that errors such as incorrect or abnormal loss of the automatic unit-conversion calculation formula and the presence of alarm codes in the analyte measurement results can all cause the failure of the LIS-based automatic unit-conversion program.

Therefore, interfaces and the unit conversion in the LIS need to be validated across the Analytical Measurement Range and should also be verified for situations where error codes are present. Additionally, it is recommended that the measurement units for the labeled values of kit calibrators should, to the greatest extent possible, align with the units commonly employed in clinical practice within the user’s country or region. When necessary, laboratory directors should appropriately convert the labeled values and units of calibrators to ensure that the analyte measurement procedures in the detection system directly report results in suitable units. Therefore, it is imperative that laboratory directors rigorously validate the measurement units when configuring analyte measurement procedures within a detection system. In the present case, adjusting the calibrator setpoints by applying a 1/100 factor to the labeled values allows the system to directly output results in g/L rather than mg/dL following recalibration. This proactive approach ensures clinical alignment and effectively eliminates the risks associated with external unit conversion within the LIS. This approach satisfies local clinical requirements and minimizes reliance on LIS-based automatic unit conversion programs. All automated calculation procedures executed within the LIS should be thoroughly documented, and all laboratory personnel must be familiar with these protocols. Risks should be effectively mitigated through regular training and comprehensive validation of data transmission.

Most clinical detection systems append alarm codes to measurement results to prompt medical laboratory professional to take appropriate actions in scenarios including: analyte concentrations exceeding or falling below the system’s dynamic range; absorbance values exceeding or falling below the detectable range; or potential interference related to sample quality (e.g., lipemia, hemolysis, icterus). In this particular case, the key issue is that the patient’s specimen had a urine total protein concentration well above the Analytical Measurement Range (given as 0–200 mg/dL) before and after the 1:2 dilution, triggering the “F” alarm code. However, the lab staff failed to recognize the meaning of the alarm code. Moreover, they did not consult the operation manual of the detection system or the laboratory-developed standard operating procedure (SOP) to understand the implication of this error code. Instead of increasing the dilution ratio of the specimen and re-testing it as prompted by the alarm code, they simply removed the alarm code in the LIS result field and reported the result. Regrettably, even when the clinician pointed out that the analyte test result might be inconsistent with the pregnant patient’s clinical manifestations, the medical laboratory professional still did not further manually dilute the specimen for testing in accordance with the requirements of the SOP. This incident underscores two critical systemic deficiencies: first, inadequate standardized training for medical laboratory professionals; and second, potential shortcomings in the laboratory’s existing competency assessment framework and performance evaluation protocols. Therefore, it is advisable that clinical laboratories implement training programs for professional technicians by integrating a patient-result-oriented modular teaching model with ISO 15189 principles and conducting periodic competency re-evaluations (4, 9). These programs should particularly focus on enhancing the training and competency evaluations not only for night-shift staff but also for new employees, those returning to work after a long-term leave, employees with position changes, and trainees undergoing training (4, 10). Furthermore, this case highlights the necessity of a robust reporting culture, less-experienced staff must be encouraged to promptly escalate anomalies, such as clinician inquiries or unresolved alarm codes, to supervisors for detailed investigation. Such proactive measures enable the laboratory to implement timely corrective actions, thereby preventing erroneous results from compromising clinical decision-making.

The Analytical Measurement Range refers to the range of reliable results achieved by analyzing an analyte via a measurement procedure or detection system, without prior preprocessing (e.g., dilution or concentration) of the sample (11). However, in clinical chemistry testing, clinical laboratories frequently encounter samples with analyte concentrations exceeding the Analytical Measurement Range–these samples require preprocessing (e.g., dilution or concentration) to bring the analyte concentration into the Analytical Measurement Range prior to measurement (12). To reduce the workload of laboratory personnel and minimize in-laboratory turnaround time, clinical laboratories frequently implement automatic dilution or concentration procedures for analytes within the detection system (11). In this case, the automatic dilution procedure for the analyte in the clinical laboratory was configured with a fixed 1:2 dilution ratio, yielding a CRR of 0–4 g/L for the detection system. Following adjustment of the dilution ratio to 1:10 on the basis of dilution recovery test results (Supplementary Table 1), the detection system’s CRR was extended to 0–20 g/L. As shown in Table 2, the percentage difference between measured and theoretical values ranged from −27.94% to −57.83% for direct measurement (no dilution), from −18.41% to −36.97% for the 1:2 automatic dilution procedure, and from −6.66% to −13.46% for the 1:10 automatic dilution procedure. This finding confirms that the configuration of dilution parameters in the automatic dilution procedure is critical: incorrect configurations directly undermine the accuracy of test results for high-concentration samples.

Matched detection systems, which comprise fixed combinations of analyzers, calibrators, and reagents produced by the same manufacturer, typically provide standardized automatic dilution or concentration procedures for use in clinical laboratories, whereas non-matched detection systems require laboratory personnel to manually configure parameters on the basis of evaluation results from dilution recovery tests. Miller et al. (12) conducted a survey of instruments accompanying 13 commonly employed urinary albumin measurement methods and found that only 6 of these methods included dilution protocols for analytes with concentrations exceeding the Analytical Measurement Range. A survey by Zhou et al. (13) highlighted that a significant proportion of clinical laboratories in China utilize non-matched detection systems for clinical chemistry testing, a situation that may be particularly prevalent. As illustrated by this case, the laboratory likely failed to establish automatic dilution parameters based on empirical dilution recovery data, which precluded the system from diluting the sample to a concentration that would suppress the “F” alarm code. Had these dilution protocols been appropriately optimized, the subsequent failure of the LIS-based unit conversion–and the resulting reporting error–could have been effectively mitigated. Therefore, we recommend that instructions for all analyte measurement procedures include recommended diluents and standardized dilution protocols. Clinical laboratories should enhance quality management for automatic dilution or concentration procedures of analytes to ensure that the CRR of the measurement procedures aligns with clinical requirements. Clinical laboratories should optimize the dilution ratios of automated measurement procedures by aligning them with empirical results from dilution recovery tests during performance verification. Simultaneously, it is imperative to enhance the proficiency of laboratory personnel in identifying and addressing platform alarm codes. Given that the nomenclature and clinical significance of these alarm codes vary across detection systems, staff must strictly adhere to the respective manufacturer’s operation manuals to ensure accurate data interpretation and handling.

This case highlights the importance of accurate transmission and standardized application of analyte units across diverse laboratory information platforms. Furthermore, it emphasizes the critical need for implementing quality assurance checks on all diluted patient samples, particularly those with high-value analytes, to ensure procedural compliance and data integrity. In summary, this case identifies significant managerial gaps in the validation of automated procedural configurations. To enhance quality assurance, it makes sense to carry out a quality assurance report check for all patient dilutions. It serves as a critical educational resource for risk assessment and quality improvement in clinical laboratories.

## 5 Conclusion

Even minor errors in the use or conversion of measurement units for any analyte test result may lead to deviations in the diagnostic direction, thereby posing a potential threat to patient safety. Therefore, clinical laboratories should place great emphasis on ensuring the consistency of measurement units for analyte test results. One recommended approach is to adopt uniform units throughout the entire workflow, including the calibration of analyte kit calibrators, the measurement output of detection systems, the release of test reports, and the application of units in clinical practice. Additionally, clinical laboratories should strengthen verification of data transmission across diverse laboratory information platforms.

## Data Availability

The original contributions presented in this study are included in this article/Supplementary material, further inquiries can be directed to the corresponding authors.
