# Supplementary material for: 24-hour urinary total protein quantitative detection for pregnant women with unit conversion failure: a case report and laboratory administration reflection
Source: Front Med (Lausanne). 2026 Mar 16;13:1763851. doi: 10.3389/fmed.2026.1763851 (PMC13033515; doi:10.3389/fmed.2026.1763851)
Supplement: Supplementary file 1 [file Data_Sheet_1.pdf]

Supplementary Table 1. Results of the 24-Hour Urine Total Protein Dilution Recovery Test

Sample No.	Pre-dilution analyte concentration （mg/dL）	Dilution ratio	Theoretical value after dilution （mg/dL）	Measured value (mg/dL)	Recovery rate (%)	PD of measured value (%)	Quality requirement for PD (%)
1[a]	184.4	1:2	94.2	98.6	104.7	4.7[b]	-15～15
2[a]	184.4	1:4	47.1	52.4	111.3	11.3[b]	-15～15
3[a]	184.4	1:6	31.4	34.6	110.2	10.2[b]	-15～15
4[a]	184.4	1:8	23.6	25.9	109.7	9.7[b]	-15～15
5[a]	184.4	1:10	18.8	20.4	108.5	8.5[b]	-15～15

Notes:

Abbreviations: PD, Percentage difference.

[a] Sample prepared by diluting 24-hour urine sample with saline at specified dilution ratios; [b] The PD between the measured value and the theoretical value met the laboratory's quality requirements.
